# Quarterly Retrospect

**Published:** 1861-10

**Authors:** 


### *Quarterly Retrospect.*

*October*, 1861.

"We have lately," says the *Times* of July 23rd, "been breakfasting full of horrors. On a glance at our columns, one might think that every possible atrocity had been committed or charged within these few weeks. If all is not true, it only follows that the imagination is quicker than the hand. It is of no use to conceal that human nature has a positive appetite for the horrible, so we will address ourselves at once to this universal infirmity. What will you have? Crimes of the worst dye lie in profusion before us. A father inflicting murderous blows on his only son! Two gentlemen shooting, hacking, and smashing one another to death in a back drawing-room in a bye street in the Strand! A surgeon charged with a professional murder upon an unhappy patient! Several husbands murdering their wives! A lady attacking her aged mother with a bludgeon! A boy stabbing his schoolfellow! A poor girl impaled by a runaway horse on the railings of Eaton-square! Several acres of conflagration, and the superintendent of the Fire Brigade and a friend crushed to death on the spot! A duke, a minister, and a bishop all dangerously ill (the two latter now, alas! dead) in the prime of life! What next? Is this *apropos* of the civil war in the States? The mail, by-the-bye, informs us that the wife of a great American poet has been just burnt to death. Was there really something 'disastrous' in the tail of the comet which they say swept the earth three weeks ago? Or is there such a thing as a run in calamity? Misfortunes, they say, never come single. *Kinglake* says, that in the Levant there are more horrors perpetrated towards the end of Lent than at any other time of the year, owing to the increase of brain disorders and morbid fancies, by inanition. But, perhaps, this is a contagion rather than an epidemic, and people's minds are just now screwed up to horror pitch by all they hear about them. There are now a dozen performers on the tight-rope, ready to perform over fountains or flames, over waterfalls or houses, and do anything that nobody else has done. There appears to be the same competition in crime."

"What is the matter with society," exclaims the *Daily Telegraph*, later in the quarter (August 5th), "what maleficent influence has overshadowed us? On every side, in every quarter of the land, we hear of deeds of horror. There seems an increase in crafty and ingenious offences, in polished knavery, in refined and smooth-faced immorality. We have reverted to first principles—to the principles of CAIN, the earliest murderer. Every day in every week, we read of some fresh crime of outrage and violence, of frenzied passion, of deliberate and sanguinary ruffianism, of gorilla-like ferocity." Here a husband murders his wife and then himself, there a wife murders her husband and then herself; here a mother poisons the whole of her children and next herself; there a child beats out his mother's brains and then hangs himself; here two lads torture a little child to death as they would have done a fly; then a soldier deliberately shoots his colonel and a captain of his regiment in the barrack-square. Add the spectacle of two crowded excursion trains rushing swiftly to destruction, and a succession of terrible conflagrations, and we complete the catalogue of horrors which have fallen upon the quarter.

"Life struck sharp on death makes awful lightning."

Amid all this confusion of horrible events, there is at least one lesson clearly to be learnt, and to this as falling entirely within our special province, we shall confine our attention. UNRECOGNISED LUNACY has played no inconsiderable part in the terrible series of murders at which we now stand aghast. We can do no better, no more useful service than bring together the different instances of murder perpetrated by UNRECOGNISED LUNATICS, which have come under the notice of our Courts of Law in the course of the past quarter. It is highly probable, from the histories of the cases, that several of those horrible murders recorded in the newspapers, which were followed by the suicide of the murderer, were the handiwork of unrecognised lunatics; but the following cases are alone sufficient, without calling upon the imagination, to show that we have here to do with a highly important, even if it be a restricted, social problem. We shall presently see, however, that Unrecognised Lunacy, even when regarded only in its criminal aspects, is not of moment solely as one of the fostering causes of murder.

It will be as well to premise, perhaps, in order to escape the too commonly perverse tetchiness of public feeling on the plea of insanity in cases of murder, that we are not recording these cases with reference to that plea. We are wishful to make plainly evident one of the most formidable evils which arises from a neglect of those signs which mark incipient disorder of the brain or the mind, and from a system of legislation which renders the early treatment of lunacy almost an impossibility.

The Summer Assizes having been held during the quarter, several o the subsequent cases bear date previous to the past three months.

MURDER.—On the 26th of March, a young woman named Diana Wickens, aged 20 years, was murdered while asleep, by her step-sister, a married woman, the wife of a sergeant-major in the 3rd Regiment of Royal Surrey Militia. The event took place under the following circumstances, at the Militia barracks, Kingston-upon-Thames. It would seem that the husband of the wretched woman, who is highly respected by his officers, had left his bed about 7 o'clock in the morning, and gone out into the barrack-yard, leaving his wife in her own bed and the murdered woman in an adjoining room, where she slept upon a temporary bed, made up for her upon a sofa, and his wife must have got up almost immediately, taken one of her husband's razors from the drawer in which it was kept, and then proceeded to the adjoining room where her step-sister was lying. There appears to be little doubt that at the time she was asleep and lying on her left side, and while in that position the murderess inflicted a dreadful wound upon the right side of the neck, dividing the right carotid artery, the jugular vein, and windpipe. Instantaneous death would not appear to have been the result, for there is very little doubt that upon the first sensation of pain the poor creature raised one of her hands, and in so doing received a severe injury upon the fingers. She then got out of bed and staggered for about six feet into the adjoining room, where she fell dead, literally covered with blood. After committing the dreadful deed the wretched woman went out to the barrack-yard, where she met a sergeant belonging to the regiment, named Oates, to whom she at once stated that she had murdered her sister, and she then went to the pump and washed her hands, which were covered with blood. To another sergeant who had hastened to the scene of the murder, on the alarm being given, she exclaimed, "I have murdered her;" and again, "Oh, I didn't do it; I couldn't do it;" and he stated that she appeared to be hardly capable of knowing what she was doing. To the policeman who arrested her, she said, "I have cut that young woman's throat, and you must take me to the station;" and at the police-station she exclaimed, exhibiting the utmost horror and repugnance at what had taken place, "This is a shocking thing I have done; I must have been insane, or I could not have done it to a sister I loved so well." The surgeon who was called in at the time, Mr. Cory, of Kingston, stated in evidence before the magistrates, that when he entered the room in which the crime had been committed, the prisoner said, "Oh God, doctor, if I had had your advice before, this would never have happened." He said also, that he had attended her in the June previous for a diseased liver. She was then very ill, but recovered, and he had advised her to keep herself quiet and free from excitement. When he saw her on the morning in question, she appeared to be in a state of dreadful excitement, her hands were cold, and her countenance was quite horror stricken. During the examination before the magistrates, the prisoner, who was a very tall, powerful, and rather plain-looking woman, seemed to be in dreadful distress of mind, and she repeatedly interrupted the proceedings by hysterical sobs and the most distressing ejaculations, saying, "Oh, it is not true! it cannot be true!"—"Oh, no, no, no! it cannot be true! oh God, oh God!"—"No, no—yes—a dream, a dream."—"Oh, it is not true, it is not true! it cannot be—it is a tale of fiction, and not a reality! If it is true, pray for me, pray for me! Oh, let me retrace my steps to my home! I am sure it cannot be!"

No motive whatever could be ascertained for the commission of the crime.

Murder (Child-).—About the termination of March (see *Times*, April 4), two children, aged respectively 5 and 8 years, were murdered by their mother, the wife of a coal-carter, at Edwardstone, near Sudbury in Suffolk, under the following circumstances:—The children were taken by her, together with an infant, a distance of half a mile, to a thick wood, where there is a large pond ten or twelve feet deep, and here the two eldest appear to have been forcibly held under water until they were drowned. The infant was destined for the same fate but some boughs stretching into the water supported it, and it was not sufficiently submerged to perish. The woman herself then walked into the water up to her neck, and, taking the children out, laid them on their faces on the grass by the side of the pond. She afterwards walked home and told a neighbour that her three children were drowned, but that she believed one was still alive. At first it was supposed that the children had met their deaths accidentally; but the woman herself subsequently made the following statement to the police constable who was called in:—"I took the little girl (the youngest and surviving child) and threw her into the pond; but she could not sink because she hung on the boughs; and as soon as I had done that, I saw the two other little dears in the pond and I rushed in after them. As soon as I got into the pond the cold water struck me, and I came to my senses and dragged the two children, now lying dead, out. The little one I took out last. When I got it out I saw it breathe." She also said she did not know which way she went to the pond or which way she came home, adding, "If I did do it, I don't know anything about it." It will be seen from this that the poor creature's language and demeanour is incoherent, and she has been, it seems, in a high state of nervous excitement for the last six months, imagining herself, without any adequate reason, to be labouring under various diseases. The younger of the two dead children had a contusion of the forehead, and the arms appeared to have been squeezed; there were no contusions on the other one, but similar marks were observable on the arms, as if they had been pressed or nipped.

MURDER (CHILD-).—OXFORD CIRCUIT.—Before Mr. Justice KEATING.—Catherine Oliver, 47, tailoress, was charged with the wilful murder of her little girl, Matilda Oliver, aged three years, on the 25th of May, 1861, at Bristol.

It appeared from the evidence that the prisoner was the wife of a poor labouring man, living at No. 4, John-street, Bristol. She had had twelve children, but six of them had died, and the charge now made against her was that she had murdered her youngest child, a little girl three years of age, by hanging her to a bedstead, on the 25th of May last. At about half-past twelve o'clock in the day a neighbour who lived opposite, as she was sitting at her window, saw the prisoner return home to her lodging with the little girl, a very nice interesting child, three years of age. At her door the prisoner took her child up in her arms and kissed her several times, and then went into the house. In about ten minutes she came out of the house alone, and having locked the door and looked into the window of the parlour in which she lodged, she went straight to the Central Police Station at Bristol, and gave herself up to a policeman there, saying, "I am your prisoner." The policeman asked, "What for?" and she replied, "For hanging one of my children." The policeman asked her what made her do that, and she said, "It is all through the ill-treatment of my husband for the last three years." She then gave the policeman a key, and said that if he took the key and would go to No. 4, John-street, he would find the child. The policeman went, as directed, and found the child in the state described, and immediately sent for medical assistance, but life was extinct. The policeman stated that the conduct of the prisoner, when she came to the station, was perfectly calm and composed, and that she maintained the same demeanour when before the coroner, and she spoke of what she had done as if it had been some matter of ordinary occurrence. The witness, Mary Shepherd, who saw the prisoner enter her house with the child, said she had known her for thirty years, and had always found her to be excessively fond of her children, and particularly of the one whose death was now in question, and that when she took the child in her arms she kissed her again and again, and then went into the house. She also said the prisoner was subject to epileptic fits, and she always considered her to be a person of very strange and eccentric habits. She was of a very religious turn of mind, and was in the habit of walking about the streets with a hymn-book, and singing hymns in a strange manner, and doing various other eccentric acts, which caused her to be called in the neighbourhood "Flighty Mrs. Oliver." She also stated that the prisoner's husband had treated her very badly for many years, and she had often seen her with bruises and black eyes. When before the coroner the prisoner made the following statement:—"I done it, sir; I am guilty, entirely through my husband's ill-conduct to me for the last sixteen years. I had three children born diseased, and one he killed before it was born. The reason I did it was because I was weary of my life, and had sooner die than live." The surgeon who had seen her in prison said, from what he saw of her, he could not say she was of unsound mind, but he thought a person who was liable to epileptic fits was likely to become insane, and sometimes that change would take place rapidly.

*Acquitted*, on the ground of insanity.

MURDER. — NORTHERN CIRCUIT, YORK.—Before Mr. Baron WILDE.—John Holdsworth, aged 37, was indicted for the wilful murder of Elizabeth Holdsworth, his wife, at Kildwick, on the 10th of June last.

The prisoner, it appeared, was the keeper of a toll-bar. He was a man of frugal habits, had accumulated from 100*l.* to 200*l.*, and certain family differences had arisen from his indisposition to aid his relations with money. On the 8th of June the prisoner's daughter went for her mother's brother, Joseph Snowden, by her mother's request, as her father, the prisoner, was unwell, and they were uneasy about him. The brother of the deceased went to the prisoner's house on Saturday, the 8th of June, and slept there that night. He heard the prisoner at six o'clock next morning call his wife up and ask her, "Lass, where's that brass?" The prisoner was also heard in the course of that day quarrelling with his wife about the bank-book which she had in her custody, and which he wanted from her. The prisoner went that night to the house of his father at Aden, and slept there, returning next morning, Monday, the 10th of June. On his return he found his wife and brother-in-law in care of the bar, and seemed annoyed at this, handing his hat to his brother-in-law, Joseph Snowden, and telling him he could do without him, he had better go. At about half-past five that afternoon, a cattle drover having passed through the gate, the deceased went out and had some dispute about the toll to be paid with the drover for cattle which had passed through the bar the day before. The brother of the deceased went out to his sister, and the drover shouted to the prisoner, and asked what he said about it. The prisoner said he could say nothing between them, as he was from home that day. The drover passed on, and whether made angry or not by the interference of his brother-in-law in the matter did not appear, but the prisoner immediately barred the half door of the toll-house, leaving his wife and Snowden outside, and handed to Snowden his hat over the half door, and told him to go. Snowden took his hat, and then told his sister, as she was going to leave her husband, she had better go away then with him. He induced his sister to go with him about 100 yards from the toll-bar on the Steeton-road, when he heard the prisoner shout from the toll-house, "Come back, lass, and let him go." The deceased then wanted to turn back, as she said he would be burning her clothes if she did not, and he returned with his sister to within 35 yards of the tollgate, when he saw a flash and heard the report of a gun, and immediately his hat was knocked off, and he saw blood on his waistcoat, and he turned and ran away. At the time he did not see his sister, but when he thought he had got out of danger he stopped, and saw his sister laid across the footpath. He ran to a house near Steeton and told a policeman. The prisoner had a double-barrelled gun hung up in his house, which witness understood was loaded. He found himself wounded in six places on the face and body. Immediately before this occurred a young gentleman named Garforth rode on a pony through the bar, and saw the prisoner in the doorway smoking a pipe, and spoke to him, saying it was a fine day. The prisoner answered, "Yes." Garforth then rode on to the house of a man named Spiers, about 150 yards off, from which the toll-bar could be seen, and there he saw the prisoner look up and down the road as Mrs. Holdsworth and Snowden were walking towards the bar, and immediately after he heard the report of a gun and saw Mrs. Holdsworth stagger and fall on the footpath, and Snowden run away. His pony galloped off with him, and he gave an alarm to a policeman whom he overtook at some distance from the bar. The prisoner was then seen to walk quickly across the road and climb over a wall, and walk quickly across the country to Aden, where he was immediately followed by the policeman and taken into custody in his father's house at Aden, and charged with the wilful murder of his wife. The prisoner said, "Yes; I've shot her;" and pointing to a beam in the house said, "hang me where I am."

When before the magistrates he made a voluntary statement—"I cannot say that I can say so much about it, for I have been fearful bad in my head. I don't know whether the gun was upstairs or down. I can remember having it in my hand when it went off, but I did not see them either inside or out. As for disturbances we have had a great deal of it lately. I cannot see what I have to leave there for, and her have plenty of money, and her and her brother about that place. They regular kept me wrong at times, like wanting me away, and I cannot see why they should. I have got nothing else to say. The way they have gone on for many a week—they wanted to turn me away without a halfpenny."

For the defence it was elicited on cross-examination of the witness Snowden, and it appeared from the learned counsel's opening for the defence and from the evidence of the prisoner's daughter, that the state of the prisoner's mind had excited the grave apprehension of his family, and had led to an arrangement being in progress for his giving up the toll-bar and going to his father's, and for the deceased to leave him and go to her relatives, as she was afraid to live with him. From infancy he had been subject to pains in his head. He was sullen, sleepless, and incessantly talked at night. Sometimes he was unable to recognise his best friends. He took irrational aversion to his wife's clothes, and to a black silk dress she had, which he called "a serpent." He was suspicious, and thought people were attempting his life, and fancied he saw wizards and devils about a Catholic priest's head. It had been frequently suggested that he should be taken to an asylum, but his wife was unwilling, and thought they could not afford the expense. There was also insanity in his family, which he might have inherited. It was elicited from his brother-in-law Snowden that he had been sent for twice because of the state of the prisoner's mind—once in May last, and two days before this event happened; that the prisoner's daughter had come for him because "her father had been carrying on very badly the night before, and they dared not stay any longer with him." He had burnt different things that night, and in consequence witness went to the bar-house on the 8th of June, to take care of his sister and niece and watch him. He went to see his brother next day, as the prisoner was rather better, and that night they persuaded the prisoner to go to his father's at Aden. About six weeks before this event the prisoner was very bad in his head, and went on so queerly he was sent to his wife's father's. While getting his breakfast the morning after he went, he said there was "something in the tea not right," and he jumped up and left the house and would not return, and his wife's father went after him, afraid that he would do himself some injury. On Sunday, the 9th of June, while the witness Snowden was at the bar-house, the prisoner took a religious tract from his hand, and read it aloud, crying. From the evidence of the prisoner's daughter it appeared that about the 1st of May the prisoner was very low-spirited, and thought everybody was trying to do him all the harm they could. He began to read his Bible, and to talk of the Catholics, and said he did not know whether he would join them, or the Ranters, or Methodists. He had never before attended a place of worship. He said he thought some copper money he had he had received from Catholics, and would not have it, but threw it across the road when it was dark. One morning he got up to take toll for a gig, and said he could not see the gig for a mist before his eyes. He thought Catholics were in the gig, and would not have the money they paid for toll, but gave it to his wife. The week after a horse galloped through the bar, and the prisoner came running downstairs, and strove to hide himself in the cellar, and she and her mother pulled him back. He then became quiet, and wished her and her mother to go out, and he would shoot himself. They sent for her uncle Thomas, and at night her father stood up in bed, and peeped through the curtains, and said her uncle wanted to hang him. He got better, but on the night of the 7th of June he kept "calling" her mother all night, telling her she was always trying to do her worst to him, and behaving badly to him. He said he thought children ran about him as they never used to do, and that the cat looked pitifully at him. He got her mother's clothes out of a drawer and attempted to burn them that night, and also a mahogany box with thread in it. In consequence, her mother wished a neighbour named Parish to come in and stay; he could not, and her mother then sent her to fetch her uncle Joseph Snowden. Her uncle next day, at her mother's request, wrote to the manager of the tolls to send another man to the gate, as her father was not fit to have the care of the gate. Her mother told him she had sent the letter, and he said then he would not leave. He had agreed before it would be better to go. He looked troubled, and cried when he read the tract on Sunday. She had hid the gun when her father was ill in May, as she thought he was not in a fit state to have it; but he had afterwards asked for it.

At the close of this witness's evidence,

His Lordship asked the learned counsel for the prosecution whether, after this evidence (which appeared to be *bonâ fide* as to steps taken by the prisoner's family before the deceased had been shot, and therefore not open to suspicion, to have him taken care of, because of his reason being disordered), a conviction could be expected.

The Counsel said, if that was his Lordship's impression, he would gladly leave the case in his hands.

His Lordship then asked the jury their opinion.

The foreman said they were unanimously of opinion that the prisoner was *Not Guilty*, on the ground of insanity.

The prisoner was then told that he would be confined during her Majesty's pleasure, and was removed from the dock.

Murder (Child-).—Midland Circuit.—Lincoln.—Before Mr. Justice Willes.—Ann Wilson, aged 37, pleaded "Not Guilty" to an indictment charging her with the wilful murder of Lucy Wilson, at Haxey, on the 9th of July last.

There were two other indictments against the prisoner, severally charging her with the murders of William and Elizabeth Wilson, which ultimately were not proceeded with, the facts of all the cases being the same.

On the morning of the 8th of July the prisoner's husband having gone to Worksop, intending to return on the 9th, the prisoner, whom he had left at home with her three children, went to the house of a near neighbour named Webster and inquired whether his little boy would take her next morning to Wheatley, the village where her father and brother lived, and where it had been arranged that her husband should call for her on his return from Worksop. Webster's wife went the same evening to talk the matter over with the prisoner, and saw the children all looking well and happy, the youngest of them sitting on the mother's knee. On the following morning Webster, in fetching the pony, twice passed the prisoner's house as early as between five and six A.M., and noticed that the windows were closed and the blinds down as if all were snug in bed. He went at six A.M. to the house and saw the prisoner alone washing herself at a sink, and inquired at what time she would be ready. She replied, whenever he was, and Webster accordingly got the pony ready, and sent his wife to inform the prisoner, who very shortly came, and was taken by the boy to Wheatley. On reaching Wheatley she was met by her brother, Joseph Parr, who inquired where she had left the children. She said she had taken care of them. He asked if she had left the nurse with them, and she answered "No." Soon afterwards he heard a cry of distress; it was from the prisoner's husband, who had met his wife and had been talking with her. He said, "She says she has put the children in the cistern." Her brother asked her if she had, and she said, "They are in Heaven!" He inquired how she did it. She told him that she fetched the youngest first, and kissed her all the way downstairs, and then fetched William and then Elizabeth. She told her father not to cry, saying that she had not done it on purpose. She wished she had them back, and asked whether they could not be brought to life again. Elizabeth, she said, cried out for help, and she (the prisoner) took up the mop to help her and pulled her up, but pulled the mop out of her hand and she splashed down again. She had always been kind and affectionate to the children, and had lived on affectionate terms with her father and brother, but latterly she had become so absent that she did not speak when addressed. On going home her story was found to be true. Her husband, in company with Webster, went to the house and found the bodies of all three children in a brick cistern, with two feet of water in it, that of Lucy being lowest. Nothing unusual had been noticed in the prisoner that morning, but it appeared that she had been confined in the previous December, and had since been very ill. Her weakness both of mind and body attracted the attention of her friends and neighbours, and by their advice she was visited by Dr. Pritchard, of Retford, who found her suffering from lowness of spirits, lost of appetite, sleeplessness, and pain in the head. He was satisfied that she was of unsound mind. Several of the witnesses for the prosecution had remarked that she was oppressed by a religious despondency and absence of mind, so that she appeared unconscious of what she was doing. She had at times a wild look, and wished that her boy was in Heaven, as he would be better done for there than she could do for him, and she should be sorry for him to grow up a fighter or a drunkard.

The learned JUDGE, addressing the jury after the evidence of Dr. Pritchard, said he did not think they could desire the case to proceed further. They must be satisfied that the prisoner was not in a sound state of mind when she did the act which was charged as murder, and believed that she was acting under a command of God higher than the law.

The jury immediately returned a verdict of *Not Guilty*.

Lack of space prevents us recording four remarkable, we might say, anomalous, cases of murder, occurring or reported in the judicial proceedings of the quarter; but we shall return to this subject and to the consideration of these cases in a subsequent number.

The following cases are worthy of being noted, as appearing in the police reports, the civil courts, and the daily press of the past three months, and further exemplifying the ills apt to arise from neglected or unrecognised lunacy.

1. A serious case of indecent assault committed by a solicitor (a married man with a numerous family), in Battersea Park, while suffering under grandiose delirium and erotic excitement, doubtless precursors of general paralysis. On the morning after his arrest, this gentleman said to one of the witnesses, "Traer, I am going to give you 1000*l.* to-morrow; Philip, 1000*l.*; and the colonel, 10,000*l.*; for I have just married a woman with 60,000*l.* a year, and we have got six carriages, and are going all over the world. I have got lots of wives—in fact, a perfect harem."

2. A case of pilfering by a young lady—a semi-imbecile. This case should never have come into a police-court. It is unjustifiable, or at the best, cruelly thoughtless, that tradesmen should place persons in a position similar to that of the accused under summary arrest, without previous inquiry.

3. A case of a well-to-do, if not wealthy, lady spending her time by driving about the streets in cabs, and then refusing to pay the owners; thus compelling them to summons her before the magistrates. She makes no answer to the charges, but simply pays the money and costs.

4. A curious case of libel, tried before Mr. Justice WILLES, at Lincoln, and of which the following is a report:—

The plaintiff is a clergyman, the incumbent of the Chapel of Langrich and Thornton. The defendant is a hawker of tea and of tracts, a teacher and a preacher, and as the learned counsel stated in his opening address, orderly, industrious, and respectable, with the exception of the uttering and publishing the slanders in question, which seemed to be founded on some strange delusion, as he must term it, although he desired to exercise the utmost forbearance towards the defendant in stating the case. It appeared that the defendant had some time previous to the spring of 1860 gone to various persons in Langrich, stating that a poor Wesleyan dressmaker who lived with her mother, and received 2s. 6d. for weekly relief from the parish, was in the family-way by the plaintiff. He stated this to the board of guardians, who in consequence discontinued the relief; and subsequently stated that she had actually been delivered of a male child. A surgeon who had attended her on account of weakness under which she was labouring, visited her for the purpose of ascertaining whether it were so, and was satisfied that she neither was in the family way nor ever had been. He met the defendant and told him so. The defendant, however, persisted in it that she had had a male child, and went to the Willertons, where he insisted, to both mother and daughter, that she had been delivered, and that Mr. Simpson was the father. These statements were mentioned to the plaintiff by a parishioner as having been said by a man who was evidently crazed, and of whose words no notice need be taken, but the plaintiff's brother hearing of the matter, wrote to the defendant, asking what he meant by making such statements of his brother. The defendant replied that he did so because they were true. In consequence of this a meeting was held of the parishioners, at which an unanimous vote was passed reprobating the defendant's conduct. Thereupon the defendant wrote the letter containing the alleged libel, which referred to the same charge, and imputed to the plaintiff that he was not fit to exercise the office of a Minister. The present action was consequently brought, and the plea which the defendent pleaded will sufficiently show with what sort of mind he had uttered these imputations. It was in these words :—

" The defendant in his own proper person for a plea to the whole declaration, says he is firmly convinced that it has been revealed to him by the Lord that the charges made against the plaintiff, as in the declaration alleged, are all true, and that the defendant spoke and wrote under the influence of that conviction, and not from malice."

This plea was withdrawn on a suggestion by the Lord Chief Justice Cockburn that it was not issuable, and a plea of *Not Guilty* and of the truth of the libel entered.

The evidence of the plaintiff, of Charlotte Willerton, of the mother, and of the surgeon entirely and completely negatived any improper connexion and the fact that Willerton had ever had a child.

The defendant was over and over again reminded by the learned judge of the groundlessness of the charges, and desired to consider whether he would not, before the verdict, retract his statements and apologize. He seemed to labour still under a hallucination, and said he could not, although he declined to address the jury or call any witnesses.

The jury found a verdict for the plaintiff, with 100*l.* damages.

